# Implementation fidelity trajectories of a health promotion program in multidisciplinary settings: managing tensions in rehabilitation care

**DOI:** 10.1186/s13012-017-0667-8

**Published:** 2017-12-01

**Authors:** Femke Hoekstra, Marjolein A. G. van Offenbeek, Rienk Dekker, Florentina J. Hettinga, Trynke Hoekstra, Lucas H. V. van der Woude, Cees P. van der Schans, ReSpAct group, Elien Heijen, Luikje van der Dussen, Anniek van Vilsteren, Jurrian van der Sijde, Henk Bosselaar, Femke van Haeften, Anke van Cuijck, Sharlon Gardeniers, Harriet Lassche, Astrid Bink, Japhet van Abswoude, Ronald van Driel, Peter van Aanholt, Treant Zorggroep, Joyce Ott-Jansen, Jacobine Schoemaker, Arno van Noord, Leo Huizer

**Affiliations:** 1 Center for Human Movement Sciences, University of Groningen, University Medical Center Groningen, PO Box 196, 9700 AD Groningen, The Netherlands; 2 Department of Rehabilitation Medicine, Center for Rehabilitation, University of Groningen, University Medical Center Groningen, Groningen, The Netherlands; 3 Healthwise, Faculty of Economics and Business, University of Groningen, Groningen, The Netherlands; 4 Center for Sports Medicine, University of Groningen, University Medical Center Groningen, Groningen, The Netherlands; 5 School of Biological Sciences, Center of Sport and Exercise Science, University of Essex, Colchester, UK; 6 Research Group Healthy Ageing, Allied Health Care and Nursing, Hanze University of Applied Sciences, Groningen, The Netherlands; 7 Department of Health Psychology, University of Groningen, University Medical Center Groningen, Groningen, The Netherlands

**Keywords:** Knowledge-translation, Multidisciplinary care, Active lifestyle, Dissemination, Mixed-methods

## Abstract

**Background:**

Although the importance of evaluating implementation fidelity is acknowledged, little is known about heterogeneity in fidelity over time. This study aims to generate insight into the heterogeneity in implementation fidelity trajectories of a health promotion program in multidisciplinary settings and the relationship with changes in patients’ health behavior.

**Methods:**

This study used longitudinal data from the nationwide implementation of an evidence-informed physical activity promotion program in Dutch rehabilitation care. Fidelity scores were calculated based on annual surveys filled in by involved professionals (*n* = ± 70). Higher fidelity scores indicate a more complete implementation of the program’s core components. A hierarchical cluster analysis was conducted on the implementation fidelity scores of 17 organizations at three different time points. Quantitative and qualitative data were used to explore organizational and professional differences between identified trajectories. Regression analyses were conducted to determine differences in patient outcomes.

**Results:**

Three trajectories were identified as the following: ‘stable high fidelity’ (*n* = 9), ‘moderate and improving fidelity’ (*n* = 6), and ‘unstable fidelity’ (*n* = 2). The stable high fidelity organizations were generally smaller, started earlier, and implemented the program in a more structured way compared to moderate and improving fidelity organizations. At the implementation period’s start and end, *support from physicians and physiotherapists*, *professionals’ appreciation*, and *program compatibility* were rated more positively by professionals working in stable high fidelity organizations as compared to the moderate and improving fidelity organizations (*p* < .05). Qualitative data showed that the stable high fidelity organizations had often an explicit vision and strategy about the implementation of the program. Intriguingly, the trajectories were not associated with patients’ self-reported physical activity outcomes (adjusted model *β =* − 651.6, *t*(613) = − 1032, *p =* .303).

**Conclusions:**

Differences in organizational-level implementation fidelity trajectories did not result in outcome differences at patient-level. This suggests that an effective implementation fidelity trajectory is contingent on the local organization’s conditions. More specifically, achieving stable high implementation fidelity required the management of tensions: realizing a localized change vision, while safeguarding the program’s standardized core components and engaging the scarce physicians throughout the process. When scaling up evidence-informed health promotion programs, we propose to tailor the management of implementation tensions to local organizations’ starting position, size, and circumstances.

**Trial registration:**

The Netherlands National Trial Register NTR3961. Registered 18 April 2013.

**Electronic supplementary material:**

The online version of this article (doi: 10.1186/s13012-017-0667-8) contains supplementary material, which is available to authorized users.

## Background

Once a health promotion program (e.g., physical activity promotion) has shown to be effective in changing individual behavior towards a healthier lifestyle, the next step is to implement the program on a larger scale [1, 2]. However, upscaling is most of the times not a straightforward process [3, 4]. Implementers have been found to especially struggle with the tension between implementing a program according to the protocol (i.e., fidelity) and adapting it to the local context [5–7]. One perspective on dealing with this ‘fidelity-adaptability’ tension is to identify pre-defined ‘core components’ of the program that are needed to be implemented strictly according to the protocol while allowing a flexible implementation of the ‘adaptable elements’ of the program [8, 9]. The assumption is that the core components are necessary to achieve the desirable program outcomes on the individual level, while adaptations will account for relevant variations in local setting and in individuals.

Implementing core components of a health promotion program in a multidisciplinary healthcare setting, such as rehabilitation care, can be complex due to the involvement of professionals with different specializations and the heterogeneous target population [10, 11]. Consequently, the extent to which core components of a multicomponent health promotion program are implemented (i.e., implementation fidelity) usually varies among organizations [12]. Moreover, implementation fidelity may also vary over time due to changes within organizations (e.g., reorganization) or changes related to involved professionals (e.g., time available to adopt the program) [13–16].

Although the importance of evaluating implementation fidelity in health promotion programs is widely acknowledged [5, 17], not much is known about the heterogeneity in implementation fidelity trajectories^1^ of national health promotion programs implemented in local multidisciplinary settings. Heterogeneity in fidelity trajectories is especially expected in multidisciplinary settings (e.g., rehabilitation care), since professionals with different roles have to work together on providing and optimizing individual patient care. Identification of different trajectories obtained from different settings (e.g., centers, hospitals), may provide directions for optimization of strategies to support implementation processes, which may subsequently contribute to the improvement of health promotion activities. Moreover, it is assumed that higher implementation fidelity is associated with better program outcomes on the individual (i.e., patient) level [18]. However, it is currently unknown whether this relationship with patient outcomes also exists in organizational-level implementation fidelity trajectories measured in a multidisciplinary healthcare context.

Therefore, the aims of this study were (1) to identify implementation fidelity trajectories of a health promotion program in a multidisciplinary setting, (2) to explore which organizational and professional characteristics are associated with these trajectories, and (3) to test whether changes in patients’ health behavior are different between these trajectories.

To gain more insight into the heterogeneity of the implementation fidelity trajectories, we used data from the nationwide implementation of the Rehabilitation, Sports and Exercise (RSE) program. The RSE program is a multicomponent physical activity promotion program in which core components are defined based on results of a previous RCT-study [19, 20]. This evidence-informed program has the goal to promote engagement in physical activities and sports in people with disabilities and/or chronic diseases during and after rehabilitation [21, 22]. Following the positive findings of the RCT-study [19, 20] conducted in 2000–2005, the RSE program was prepared for scaling up to more settings in order to reach a broader population. During a three-year nationwide approach (2013–2015), the RSE program was implemented in different rehabilitation settings across the Netherlands. A longitudinal design was used to evaluate the implementation of the RSE program on organizational- and patient-level, making it an exemplary case [23] to study implementation fidelity trajectories in a multidisciplinary setting.

### Theoretical and practical contributions

This study has theoretical and practical contributions. To the implementation fidelity literature (e.g., [17, 18]), the findings are expected to add insight on the impact that differences in organizational-level implementation fidelity trajectories may have on ultimate patient behavior studied in complex settings (i.e., rehabilitation) and in a heterogeneous population (i.e., disabled persons). Moreover, this study offers possible explanations for the heterogeneity in implementation fidelity trajectories of a health promotion program in multidisciplinary context, in terms of tensions that are managed differently across settings. The identified implementation fidelity trajectories and the associated organizational and professional characteristics may support implementers (e.g., healthcare professionals, policy-makers, managers) in making more informed implementation decisions for health promotion programs. In other words, it provides directions for what kind of assistance (i.e., implementation strategy) may be effective in different settings (e.g., large vs small organizations) when scaling up national health promotion programs to local multidisciplinary settings.

### Conceptual framework

The conceptual framework described by Wierenga et al. (2013) [24] was used as guide for the design of the current study [22]. This framework builds upon and integrates earlier frameworks and models [14, 25–28]. The framework includes the following ingredients:

- Three phases of an innovation processes: adoption, implementation, continuation;
- Five domains of determinants influencing the innovation process: socio-political, organization, professional, program, patient;
- The implementation strategy.

The organizations participating in our study received support to implement the RSE program during a 3-year period (2013–2015), which we defined as the implementation phase. The continuation phase (i.e., sustainability) started after the program period (January 2016). We used Wierenga et al.’s classification of the determinants (e.g., socio-political, organization, professional) for the description of the identified trajectories during the implementation phase. Based on Hoekstra et al. (2017) [29], we assumed that two domains of determinants, namely, the organization and professional, varied the most across the participating organizations. For the purpose of the current analysis, we therefore specifically focused on variance in determinants related to the organization and the professional. Lastly, the activities initiated by the national program coordinators that were part of the implementation strategy were mainly the same across the participating organizations and are described in the Methods section below.

## Methods

### Design of the study

The analyses were based on data from the Rehabilitation, Sports and Active Lifestyle (ReSpAct) study, which is a multicenter longitudinal study designed to evaluate the RSE program on organizational- and patient-level [21, 22]. Survey data filled in by rehabilitation professionals were used to assess implementation fidelity in 17 organizations at three moments in time. Different methods (online registration system, surveys, logbooks, interviews) were used to collect information about organizational and professional characteristics. Patient survey data from the baseline and the first follow-up measurement were used to obtain information about the program outcomes on patient-level (changes in patients’ physical activity behavior).

### Setting and study population

Implementation fidelity was assessed at 17 locations consisting of 12 rehabilitation centers and 5 rehabilitation departments of hospitals. Inclusion criteria for these organizations were (1) willingness to implement and continue the RSE program, (2) willingness to support the ReSpAct-study, (3) being involved in the implementation of the RSE program during the whole program period.

The program coordinators (i.e., program owners) initiated and coordinated the implementation process in the participating organizations on a national level. Information about organizations’ adoption process and implementation strategy was obtained from logbooks of the program coordinators (*n* = 2).

Rehabilitation professionals (managers, physicians, project leaders, counselors) provided information about the implementation process in their organization. Inclusion criteria for professionals were (1) being actively engaged in the implementation of the RSE program, (2) working at the location of the organization that received financial incentive for implementing the program. All professionals meeting the inclusion criteria were asked to participate in the study by filling in a survey at three points in time. In each organization, a project leader and at least one counselor had been appointed to implement and execute the program. In some situations, one professional fulfilled multiple roles (e.g., manager and project leader).

All adult patients participating in the RSE program were asked by the counselors to participate also in the linked ReSpAct-study [21]. Data from participating patients were used to investigate changes in physical activity behavior between baseline and first follow-up. Inclusion criteria were (1) being 18 years and older, (2) having a physical disability and/or chronic disease, (3) receiving an outpatient rehabilitation treatment in one of the selected locations of the participating organizations, (4) participating in the RSE program.

### The rehabilitation, sports and exercise program

The evidence-informed RSE program consists of six core components [22]:

1. Patients receive an intake session with a rehabilitation professional (e.g., physician, physiotherapist) to discuss their interests in participation in sports and exercise activities during their rehabilitation treatment.
2. Patients take part in sports and exercise activities during rehabilitation.
3. Patients are referred to the Sports Counseling Center (SCC) at the end of their rehabilitation treatment.
4. Patients receive tailored advice on active lifestyle during a face-to-face consultation at the SCC by using motivational interviewing (MI) to initiate a behavioral change [30].
5. Patients are provided with four telephone-based counseling sessions initiated by counselors working in the SCC to further stimulate patients in maintaining an active lifestyle after rehabilitation.
6. The counselors working in the SCC collaborate with sports and exercise providers in the community.

The organizations (*n* = 17) received support to implement the RSE program in their daily routines. The support consisted of national and regional meetings for involved professionals, advice and support from program coordinators, financial incentives, provision of promotion material, and educational courses in MI.

### Data measures and instruments

#### Implementation fidelity scores

Information about the implementation fidelity was collected by survey data. Professionals with different roles were asked to fill in a survey at three time points (T0: April 2013, T1: June 2014, T2: September 2015). The survey contained questions about the extent to which the core components of the RSE program were implemented in the organization. Completing the survey took approximately 30 to 40 min. Surveys were adapted to the role of the professional (manager, project leader, physician, counselor) indicating that the survey included questions associated with the tasks of the professionals.

Implementation fidelity was measured by calculating a total fidelity score (%) for each organization at each time point (T0, T1, T2) [12]. The fidelity scores were calculated using a selection of closed-ended questions from the professionals’ surveys that specially focused on the six core components of the RSE program (see Table 1). Next, answers were dichotomized into ‘yes’ if the answer on the question was in line with these predefined core components. For each time point (T0, T1, T2) and for each organization (*n* = 17), a total fidelity score was calculated by counting the number of questions dichotomized into ‘yes’ and dividing it by the maximum score (T0: *n* = 12, T1: *n* = 11, T2: *n* = 12). Total fidelity scores are presented in percentages in which higher scores are associated with a more complete implementation of the core components of the RSE program.

**Table 1:** Core components and related items used to assess organizational-level implementation fidelity

Core components and related items	Moment of measurement	Source
1. Intake session on exercise and sports		
▪Takes place	T0, T1, T2	PL
▪As standard component of rehabilitation^a^	T0, T1, T2	PL
2. Exercise and sports during rehabilitation		
▪‘Sports and exercise during rehabilitation’ is part of the official policy of the organization	T0, T2	M
▪ The topic ‘sports and exercise’ is discussed during a multidisciplinary team meeting^b^	T0, T1, T2	Ph
3. Referral to SCC		
▪ Takes place	T0, T1, T2	PL
▪ Is a standard component of rehabilitation^a^	T0, T1, T2	PL
4. Face-to-face consultation		
▪Is a standard component of rehabilitation^a^	T0, T1, T2	PL
▪All counselors use MI during almost every consultation	T0, T1, T2	C
5. Counseling sessions		
▪ Takes place	T0	PL
▪Is a standard component of rehabilitation^a^	T0, T1, T2	PL
▪Takes place according to the guidelines^c^	T1, T2	C
6. Collaboration between SCC and external sports and exercise facilities		
▪Collaboration between SCC and external exercise and sports facilities	T0, T1, T2	C
▪All counselors have knowledge of sports and exercise facilities in the region	T0, T1, T2	C

Note. ^a^1 point if it is a standard component for (almost) all outpatients or for only some groups of outpatients, 0 point if it is not a standard component at all or I do not know. ^b^1 point if it is discussed always or most of the time, 0 point if it is discussed never or sometimes. ^c^1 point if all counselors never or sometimes deviate from the guidelines, 0 point if all or some counselors often or most of the times deviate from the guidelines

*PL* project leader, *M* manager, *Ph* physician, *C* counselor, *MI* motivational interviewing, *SCC* Sports Counseling Center

#### Professional and organizational characteristics—surveys

Information about professional (appreciation, support) and organizational characteristics (compatibility, staff turnovers, financial resources) was derived by collected data from the surveys filled in by professionals at the start (T0) and end (T2) of the program period. Questions were closed-ended, answered on a 4 or 5 Likert scale.

#### Professional and organizational characteristics—interviews

Qualitative data from interviews conducted with rehabilitation professionals and program coordinators were used to verify the quantitative data and identify additional relevant characteristics. Researcher FH (first author) invited rehabilitation professionals (i.e., all project leaders and a selection of the involved counselors) for a semi-structured interview. During these interviews, open questions were posed about perceived facilitators and barriers to the implementation and continuation of the RSE program in their organization. The interviewer probed for the organization’s plans and procedures concerning the implementation process.

Researcher FH invited the program coordinators for two separate interviews. In the first interview, open questions were asked about their experiences with the program’s implementation in each participating organization. The second interview focused on program coordinators’ perceptions of factors influencing the implementation process. Further details about the data collection procedures in the interview rounds are described in Hoekstra et al. (2017) [29].

#### Patients’ physical activity behavior

Information about patients’ outcomes was collected by survey data from patients enrolled in the ReSpAct-study. The baseline measurement took place between 3 and 6 weeks before the end of the outpatient rehabilitation treatment and the follow-up measurement took place 14 weeks after the end of outpatient rehabilitation. Patients’ physical activity behavior at baseline and follow-up were measured with the adapted version of the Short QUestionnaire to ASses Health enhancing physical activity (SQUASH) [21, 31]. The original SQUASH has been shown to have an acceptable validity and test-retest reliability in health individuals and in specific patient groups [31–33]. Based on the answers of the SQUASH, a physical activity score, which is a combination of duration and intensity of all reported physical activities, was calculated for each patient at baseline and follow-up. The physical activity score included an age-related correction. The change in physical activity behavior was calculated by subtracting the physical activity score at baseline from physical activity score at follow-up. The surveys included also questions about general demographical information, patients’ psychosocial status, and perceived barriers to physical activity [21].

### Data analyses

Data analyses consisted of four main steps. Firstly, an agglomerative hierarchical cluster analysis based on Ward’s method [34] with a squared Euclidian distance measure was conducted to gain insight into the variation of implementation fidelity trajectories. This cluster analysis was used to identify clusters of organizations with a minimum within-cluster variation and a maximum between-cluster variation in total fidelity scores at different time points. The number of clusters was decided based on the agglomeration schedule coefficient, the dendogram, and on visual inspection of the different cluster solutions [35].

Secondly, Mann-Whitney *U* tests were performed to assess differences between the clusters of organizations in implementation determinants in order to externally validate the clusters and explain differences between the clusters. These determinants were related to the professionals (e.g., support, appreciation) and the organization (i.e., awareness of SCC within organization, financial resources) measured at the start (T0) and end of the program period (T2). Determinants were selected using the outcomes of a previous qualitative study on perceived facilitators and barriers to the implementation of the RSE program [29]. Determinants were selected if they were measured in the T0 and T2 surveys *and* if they had been experienced as barrier or facilitator by professionals in different organizations in the previous qualitative study [29].

Thirdly, qualitative data derived from the interviews were used to verify and interpret the quantitative data using triangulation. All interviews were recorded and transcribed as described previously [29]. All transcripts were independently coded by two coders following an open coding procedure. Discrepancies in coding procedures were discussed in order to reach consensus. The coding scheme that was developed and used in this study contained codes for potential facilitating and hampering factors. Afterwards, codes were clustered into broader factors and assigned to one of the domains of the theoretical framework (socio-political, organization, professional, program, patient). For the purpose of the current study, the first author (FH) re-read and re-analyzed the coded transcripts using ATLAS.ti (ATLAS.ti Scientific Software Development GmbH, Berlin, Germany). The analyses were specifically focused on analyzing the differences in professional and organizational characteristics between the identified clusters. FH, who collected and analyzed the data, selected key differences and discussed the findings with MAGO, who has an expertise in change management processes in multidisciplinary healthcare settings. Afterwards, the other authors, with diverse expertise (e.g., rehabilitation, physical activity promotion, sports and health, disability, epidemiology) reflected on the findings.

Lastly, linear regression analyses (crude and adjusted models) were conducted to test whether changes in patients’ physical activity behavior were associated with organizational fidelity on basis of the identified clusters. The adjusted model was corrected for the following confounders: gender, stage of change at baseline, stage of change in the past, motivation, self-efficacy at baseline, number of received telephone-based counseling sessions, and the extent to which patients’ disability/disease impede an active lifestyle. Confounders were chosen based on the procedure described by [36]. Statistical analyses were performed using SPSS version 20.0 (SPSS Inc., Chicago, IL, USA). The statistical significance level was set on *p* < .05.

## Results

Professionals’ response rates on the T0, T1, and T2 surveys were respectively, 94, 86, and 88% (Table 2). Response rates were highest for the project leaders and counselors.

**Table 2:** Professionals’ response rates to the three surveys

Professionals	T0	T1	T2
Manager	11/12 (92%)	10/13 (77%)	11/14 (79%)
Project leader + manager	6/6 (100%)	3/3 (100%)	5/5 (100%)
Project leader	9/9 (100%)	10/10 (100%)	6/6 (100%)
Project leader + counselor	4/4 (100%)	4/4 (100%)	6/6 (100%)
Counselor	26/28 (93%)	21/25 (78%)	23/23 (100%)
Physician	13/14 (93%)	11/14 (79%)	15/21 (71%)
Total	69/73 (94%)	59/69 (86%)	66/75 (88%)

Note. A 100% response rate indicates that all professionals that were asked to fill in the survey, completed the survey in that specific round (T0, T1, T2)

In addition, all professionals invited for a semi-structured interview agreed to participate. A total of 23 interviews were conducted with rehabilitation professionals (*n* = 27) and program coordinators (*n* = 2) between October 2014 and April 2015. Interviews were conducted with one professional or with two professionals (i.e., single or double interview design). Details about professionals’ roles and interview designs are shown in Appendix 1.

### Implementation fidelity trajectories

**Fig. 1:**
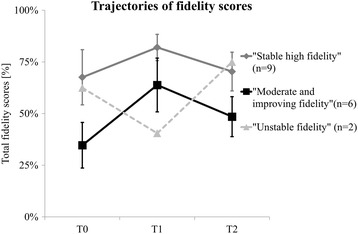
Three clusters of organizations with different implementation fidelity trajectories

Based on the results of the hierarchical cluster analysis, three clusters of organizations (*n* = 17) were identified: stable high fidelity (*n* = 9), moderate and improving fidelity (*n* = 6), and ‘unstable fidelity’ (*n* = 2). Figure 1 shows the total fidelity scores for each cluster over time. The ‘stable high fidelity’ cluster consisted of five rehabilitation centers and four rehabilitations departments of hospitals and the moderate and improving fidelity cluster consisted of six rehabilitation centers. The unstable fidelity cluster consisted of one center and one hospital. Because of the small sample size (*n* = 2), this cluster was not included in the next steps of analyses, in order to maintain anonymity. Mean ± SD fidelity scores of the two largest clusters were highest halfway program period (stable high fidelity cluster: T0: 68% ± 13%, T1: 82% ± 6%; T2: 70% ± 9%; moderate and improving fidelity cluster: T0: 35% ± 11%, T1: 64% ± 13%; T2: 49% ± 10%).

Table 3 describes the general characteristics of the stable high fidelity and moderate and improving fidelity clusters. The stable high fidelity cluster contained relatively smaller organizations and more early starters compared to the ‘moderate and improving fidelity’ cluster. Professionals’ response rates to the surveys tended to be lower in the ‘moderate and improving fidelity’ cluster.

**Table 3:** General characteristics of the identified clusters of organizations

General characteristics of the organizations	Cluster 1 Stable high fidelity (*n* = 9)	Cluster 2 Moderate and improving fidelity (*n* = 6)
Setting		
Center/hospital	44.4% (4)/55.5% (5)	100.0% (6)/0.0% (0)
Size of organizations		
Small/large	77.8% (7)/22.2% (2)	33.3% (2)/66.7% (4)
Implementation started before T0 measurement		
Yes/no	44.4% (4)/55.6% (5)	16.7% (1)/83.3% (5)
Professionals’ response rates to surveys		
T0 (median + IQR)	100% + 7%	100% + 17%
T1 (median + IQR)	100% + 22%	78% + 29%
T2 (median + IQR)	100% + 20%	79% + 35%
Staff turnover of manager, project leader or physician		
Between T0 and T1 (% yes)	44% (4)	33% (2)
Between T1 and T2 (% yes)	44% (4)	67% (4)
Continuation of RSE program after implementation period		
Yes	88.9% (8)	83.3% (5)

Appendix 2 describes the fidelity components at T0 and T2 for each of the two main clusters. At T0, stable high fidelity organizations were more likely to execute an intake session, refer patients to the SCC, provide counseling after rehabilitation, and collaborate with external sport and exercise providers. At T2, stable high fidelity organizations were more likely to implement a referral to the SCC, a face-to-face consultation, and counseling after rehabilitation as a standard component of the outpatient rehabilitation treatment.

### Professional and organizational characteristics

Although professionals were generally positive about the implementation of the RSE program, the levels differed between both clusters (Table 4). At the implementation period’s start and end, support from physicians and physiotherapists, professionals’ appreciation, and program compatibility were rated more positively by the professionals working in stable high fidelity organizations as compared to the moderate and improving fidelity organizations (*p* < .05, see Table 4). Moreover, managers and project leaders working in the stable high fidelity organizations were more positive about the *financial resources* available to execute the RSE program as compared to those in moderate and improving fidelity organizations.

**Table 4:** Differences between clusters in professional and organizational characteristics at the start and at the end of the implementation period

	Cluster 1 Stable high fidelity		Cluster 2 Moderate and improving fidelity		Differences	
	Mean (SD)	*N*	Mean (SD)	*N*	*U* or *Z* values	*p* values
Start of implementation period (T0)						
Characteristics of professionals						
Support from other professionals^a^						
Management	4.2 (0.9)	37	4.3 (0.6)	24	*z* = − 0.01	*p* = .994
Rehabilitation physicians	4.4 (0.6)	37	3.9 (0.5)	24	*z* = − 2.91	*p* = .004*
Physiotherapists	4.5 (0.8)	37	4.0 (0.8)	25	*z* = − 2.91	p = .004*
Sports therapists	4.8 (0.5)	30	4.7 (0.5)	25	*z* = − 1.25	*p* = .210
Counselors	4.9 (0.3)	32	4.7 (0.4)	23	*z* = − 1.64	*p* = .102
Professionals’ appreciation^b^	8.3 (0.7)	31	7.9 (0.9)	22	*z* = − 2.61	*p* = .009*
Characteristics of the organization						
Compatibility of program in organization^a^	4.5 (0.6)	37	3.8 (0.9)	25	*z* = − 2.993	*p* = .003*
End of implementation period (T2)						
Characteristics of professionals						
Support from other professionals^a^						
Management	4.3 (0.6)	34	3.9 (0.9)	19	*z* = − 1.60	*p* = .110
Rehabilitation physicians	4.5 (0.6)	37	3.8 (0.9)	20	*z* = − 3.20	*p* = .001*
Physiotherapists	4.6 (0.5)	37	3.9 (0.9)	22	*z* = − 3.03	*p* = .002*
Sports therapists	4.9 (0.3)	28	4.6 (0.7)	21	*z* = − 1.96	*p* = .050*
Counselors	4.9 (0.4)	35	4.7 (0.5)	20	*z* = − 1.39	*p* = .165
Professionals’ appreciation^b^	8.5 (0.8)	37	7.9 (0.9)	22	*z* = − 2.24	*p* = .025*
Characteristics of the organization						
Awareness of SCC within organization^a^	3.9 (0.8)	37	3.4 (0.9)	22	*z* = − 1.88	*p* = .061
Compatibility of program in organization^a^	4.6 (0.6)	36	3.1 (1.0)	22	*z* = − 5.19	*p* < .001*
Sufficient financial resources to execute the program in a satisfactory way^c^	4.1 (1.1)	16	3.0 (1.2)	8	*U* = 30.5	*p* = .038*

Notes. ^a^Measured on a Likert scale: 1 = very bad to 5 = very good, ^b^measured on a 10-point scale, ^c^measured on a Likert scale: 1 = strongly disagree to 5 = strongly agree, *SCC* Sports Counseling Center, *SD* standard deviation. * Statistical significant (p<.05) difference between both clusters. Means instead of medians were presented in order to illustrate the direction of the differences

Moreover, the referral procedure from patients to the SCC was significantly different between both clusters (Table 5). Patients from stable high fidelity organizations were more often referred to the SCC by rehabilitation physicians (30.5 vs 13.6%) or the multidisciplinary team (16.5 vs 0%), while patients from moderate and improving fidelity organizations were more often referred by a sport therapist or physiotherapist.

**Table 5:** Characteristics of patients in stable high fidelity and moderate and improving fidelity organizations

Patients’ characteristics	Cluster 1 (C1) Stable high fidelity	Cluster 2 (C2) Moderate and improving fidelity
Age* mean (SD) (C1: *n* = 843, C2: *n* = 415)	49.0 (13.2)	51.3 (14.0)
Gender* % (N) (C1: *n* = 844, C2: *n* = 417)		
Female	56.9% (480)	50.1% (209)
Diagnose* % (N) (C1: *n* = 831, C2: *n* = 412)		
Brain disorders (e.g., stroke)	27.6% (229)	29.1% (120)
Disorders of locomotor system	20.6% (171)	14.3% (59)
Chronic pain	19.0% (158)	15.8% (65)
Neurologic disorders	6.6% (138)	9.5% (39)
Disorders of organs	5.4% (45)	19.4% (80)
Other disorders (e.g., amputation, spinal cord injury)	10.8% (90)	11.9% (49)
Number of received counseling sessions (telephone and email)* (C1: *n* = 844, C2: *n* = 417)		
0 e-mails/phone calls	18.0% (152)	7.9% (33)
1–3 e-mails/phone calls	62.2% (525)	44.8% (187)
4 or more mails/phone calls	19.8% (167)	47.2% (197)
Referred to SCC by:* (C1: *n* = 678, C2: *n* = 345)		
Rehabilitation physician	30.5% (207)	13.6 (47)
Sport therapist	29.8% (202)	32.2% (111)
Physiotherapist	22.3% (151)	53.6% (185)
Multidisciplinary team	16.5% (112)	0% (0)
Other	0.9% (6)	0.6% (2)
Physical activity behavior at baseline (C1: *n* = 755, C2: *n* = 385) and follow-up (C1: *n* = 573, C2: *n* = 273)		
Physical activity score at baseline (median + IQR)	3300 + 5024	3420 + 5963
Physical activity score at follow-up (median + IQR)	2940 ± 4968	2958 ± 4915

Notes. Stage of change at baseline and physical activity levels are obtained from survey-data filled in by patients. Other patients’ characteristics are obtained from the online registration system filled in by counselors. *Statistical significant (*p* < .01) difference between both clusters based on chi-square tests. *C1* cluster 1, *C2* cluster 2, *SCC* Sports Counseling Center

As presented in Table 6, the qualitative data confirmed the abovementioned differences between both clusters of organizations. Program coordinators reported that before the start of the program period, several physicians in the stable high fidelity organizations pointed out their interests in the RSE program illustrating their proactive roles towards the implementation process. Another remarkable finding was that stable high fidelity organizations had often an explicit vision and strategy about the implementation of the program in their organization. Accordingly, these professionals seem to be more creative in adapting the program to their local context (see example quotes in Table 6).

**Table 6:** Key differences based on interviews with program owners and professionals

Stable high fidelity cluster (*n* = 9)	Moderate and improving fidelity cluster (*n* = 6)
*Adoption period* ^a^	
Organization’s starting position^b^ was high (*n* = 3), moderate (*n* = 4) or low (*n* = 2)	Organization’s starting position was high (*n* = 2) or low (*n* = 4)
-The organizations (*n* = 2) with low starting positions improved within a short period	-The organizations (*n* = 4) with low start positions improved within a moderate to long period
“They [professionals in one organizations] prepared the implementation [of the program] within 3–4 weeks. This illustrates their fast improving ambition levels.” [Quote from a program coordinator]	“It was a very difficult starting process, because of the many staff-turnovers at management level.” [Quote from a program coordinator]
Ambition level during adoption	Ambition level during adoption
-High ambition level (*n* = 6)	-High ambition level (*n* = 2)
-Ambition level was not discussed (*n* = 3)	-Moderate to low ambition level (*n* = 3)
	-Ambition level was not discussed (*n* = 1)
*Implementation period* ^a^	
Role of physicians	Role of physicians
-Proactive role before the start (*n* = 4) -Active engagement during implementation (*n* = 9)	-No or less active engagement before and during the implementation (*n* = 6)
“In 2011, we presented our Handbook at a national meeting organized for rehabilitation physicians. Afterwards, he [a physician of a participating organization] came to me and said ‘I really want to have that Handbook, because I want to implement that program’ [RSE].” [Quote from a program coordinator]	“It was a conscious choice. […] At the start of the project, we were in the middle of a re-organization. And during that time, we were understaffed. And we tried to involve a physician, but it didn’t worked out.” [Quote from a project leader and counselor] “We have to start a project, and none of the physicians had time [to be member of the work group]. […] And that’s why one of the physicians was involved from the background, as a sounding board for me. […] But nobody participates in the work group. [Quote from a project leader]
Changes in organizations	Changes in organizations
-The impact of staff turnover processes was not explicitly discussed during interviews (*n* = 9)	-Staff turnover processes delayed the implementation (*n* = 2)
-Reorganizations took place (*n* = 1)	-Reorganizations took place (*n* = 3)
Organization’s vision and strategy	Organization’s vision and strategy
-The majority (*n* = 8) had an explicit vision and strategy about the implementation of the program	-The minority (*n* = 1) had an explicit vision and strategy about the implementation of the program.
“They implemented a standardized group-based intake session [of the program]. At the start of the rehabilitation treatment, all patients receive a group-based intake session about sport and exercise opportunities.” [Quote from a program coordinator]	“Eventually, I mainly used the Handbook [of the program] to write the project plan. [..]. That [Handbook] was a very useful tool.” [Quote from a project leader and counselor]

Notes. ^a^Information about the adoption period is mainly derived from the interviews with the program coordinators. Information about the implementation period is derived from interviews with the program coordinators and involved professionals (project leaders, managers, and counselors). ^b^Organization’s starting position refers to the extent to which organizations had already implemented components of the program within their daily routines during the adoption period

### Patients’ outcomes

Patients’ baseline characteristics (age, gender, diagnosis, stage of change) enrolled in the ReSpAct-study were significantly different between patients from stable high fidelity and moderate and improving fidelity (see Table 5, *p* < .05). In addition, relatively more patients from the moderate and improving fidelity organizations received the complete counseling protocol (i.e., four or more sessions) compared to patients from the stable high fidelity organizations (47.2 vs 19.8%, Table 5).

The crude and adjusted regression analyses showed no significant difference in changes in physical activity scores between patients from the stable high fidelity and moderate and improving fidelity organizations (crude model: *β* = − 789.5, *t*(786) = − 1.587, *p* = .113; adjusted model: *β =* − 651.6, *t*(613) = − 1032, *p =* .303).

## Discussion

We used a new approach to generate insight into the heterogeneity of implementation fidelity trajectories of a health promotion program in multidisciplinary setting. Moreover, we showed how these implementation fidelity trajectories were associated with changes in patients’ health behavior. Our insights were based on longitudinal data of the nationwide implementation of an evidence-informed physical activity promotion program in Dutch rehabilitation care.

### The implementation fidelity trajectories

The stable high fidelity and moderate and improving fidelity organizations showed a trajectory in which fidelity scores were highest halfway the implementation period. Since conceptualizations of implementation fidelity vary [5, 37, 38] and nationwide longitudinal health promotion implementation studies are relatively scarce, direct comparison with other studies is difficult. Two studies of a multicomponent health promotion program also reported decreasing implementation fidelity over time [39, 40]. Another study in an educational setting, however, showed how implementation fidelity can improve over time as professionals gain experience [41]. The modest decline in implementation fidelity in our sample is in line with the diffusion of innovation theory [27], which predicts a decrease in implementation fidelity as a result of local adaptations (or ‘reinventions’).

Our fidelity measures related to the core components of the RSE program that were assumed to be required for sustainable integration of physical activity promotion during and after rehabilitation. We had expected adaptations both within and beyond these core components (e.g., mail counseling instead of or additional to telephone-based sessions) in order to adjust to local conditions (e.g., different patient characteristics) and establish an optimal ‘fit’ between the program and context [8, 42]. As our operationalization incorporated such adjustments, we had expected an improvement of implementation fidelity over time. Still, the majority of the organizations showed a fluctuating trajectory (i.e., increasing and decreasing fidelity scores).

We may explain these three fluctuating trajectories as follows. Achieving high fidelity for the health promotion program required engagement of professionals with different roles. Fluctuations may have occurred as a result of changes in socio-political (e.g., aborting financial incentives), organizational (e.g., staff turnovers), and professional factors (e.g., engagement levels) [42]. In our organizational-level analysis, a 100% fidelity score reflects an integrated use of all program components within the rehabilitation service offering, which is assumed to make the program resilient to the aforementioned disturbances. Even though none of the organizations achieved a 100% fidelity score, almost all sustained the program. This finding supports Chamber’s et al. (2013) [42] principle of dynamic program implementation and execution being conditional for sustainability. Durlak [43] already proposed a minimum threshold for implementation fidelity leading to effective and sustainable health promotion. Future research may gain more precise insight into variation in ‘threshold’ values over time for different program types and settings.

### Organizational and professional characteristics

The results showed that stable high fidelity organizations were generally smaller, started earlier, and implemented the program in a more structured way compared to moderate and improving fidelity organizations. These findings are in line with earlier studies showing that implementation is easier in smaller organizations [14, 24]. Furthermore, it was paradoxical that stable high fidelity organizations showed more adaptations than moderate and increasing fidelity organizations. The higher adaptation rate among stable high fidelity organizations might be partly explained by the fact that these organizations adopted the program earlier in time. Simultaneously, the early start afforded the professionals in the stable high fidelity organizations a longer period to implement the RSE program, resulting in generally higher fidelity scores at all time points.

Moreover, the results showed professionals’ positivism and support for program implementation to be highest in stable high fidelity organizations. These results accord with reviews that find professionals’ attitudes, support from colleagues, and program compatibility to positively influence organizational-level implementation of (health promotion) programs [13–16].

### Tensions in implementing health promotion programs in multidisciplinary settings

Our analysis of the differences in organizational and professional characteristics between the stable high fidelity and moderate and improving fidelity trajectories, points towards three tensions that need to be managed when implementing health promotion programs in multidisciplinary settings (cf. [44, 45]).

The *first tension* that arose concerned the dichotomy between implementing according to the nationwide evidence-informed protocol and according to the local organization’s health promotion vision. The former is an implementation fidelity requirement [18], whereas the latter is a critical change management requirement [46]. Our findings demonstrate how the stable high fidelity organizations had more often an explicit own vision and strategy about the program’s implementation than the moderate and improving fidelity organizations. Theoretically, such a change vision supports the contextualization of an innovation, i.e., the health promotion program, which is required for its successful implementation [47]. A change vision directs the alignment of an innovation with the organization’s procedures and routines, which contributes to its sustainability [42, 46]. Therefore, paradoxically, stable high fidelity organizations showed more adaptations than moderate and increasing fidelity ones. Thus, in the first cluster, contextualization and alignment have somehow been reconciled with the guaranteeing of sufficient implementation fidelity to afford the desirable health outcomes on patient-level [5, 18]. Based on these findings, we propose that determining the core components of the concerning program and integrating these core components within the organization’s change vision helps implementers to overcome the ‘fidelity-vision’ tension. By showing the role of the organization’s change vision in achieving high implementation fidelity, the fidelity-vision tension extends earlier research on the ‘fidelity-adaptability’ tension [5, 6, 17, 48].

The *second tension* we came across was the balancing between physicians’ active engagement and management’s buffering of scarce physician resources. On the one hand, engagement of the most influential professionals, i.e., physicians, helps implementation, and sustenance [46, 49]. On the other hand, the active involvement of different professionals (e.g., physician, physical therapists, sports counselors) is time intensive and costly. Our study adds to this literature, by our results’ suggestion that the balancing largely depended on the organization’s size. In small organizations, which were more represented in the stable high fidelity cluster, active engagement of physicians was found and seems to have positively contributed to implementation. In contrast, in large organizations, which were more represented in the moderate and improving fidelity cluster, physicians were not actively engaged. As most organizations in both clusters sustained the program after its implementation, both strategies seem feasible, yet only in different settings (large- vs small-sized organizations). Besides the organization’s size, the current organizational circumstances seem also important for managing this tension. In the context of a re-organization, regardless of size, management’s buffering of physician resources seems most feasible. Still, a balance needs to be maintained: as physician engagement coincided with stable high fidelity and was found crucial for longer term sustainability [29], the extent to which the active engagement of key professionals can be traded off against their relatively scarce time and high costs remains limited.

The *third tension* involves the balancing between the choice for a high fidelity implementation and for a cost-efficient implementation strategy. The stable high fidelity organizations feature a strategy aimed at *high fidelity implementation*, while our data suggest that the ‘moderate and improving fidelity’ organizations stressed *cost-efficient implementation*. Consequently, the moderate and improving fidelity organizations achieved comparatively lower levels of implementation fidelity, yet these organizations showed higher continuous improvement in implementation fidelity over time. Apparently, an incremental implementation trajectory was more affordable and moderate fidelity achieved over the study’s time span, did not result in lower patient outcomes than in the stable high fidelity cluster. On the contrary, the routinization of the program components was lower in the moderate and improving fidelity organizations suggesting less promising results regarding the sustainability of the program fidelity on the longer term. These insights are relevant for investors who want to implement and scale up their health promotion programs. They should make decisions about how they want to invest their money and how to deal with a trade-off between quality and efficiency?

Appendix 3 summarizes how the three tensions relate to the different implementation fidelity trajectories. The findings illustrate how tensions are managed differently under different circumstances and settings suggesting that different strategies seem feasible in different settings (e.g., small- vs large-sized organizations) and circumstances (e.g., low and high starting positions). These insights are relevant when scaling up health promotion programs to local settings; it illustrates the need to apply a (more) tailored implementation strategy depending on organization’s starting positions, organization’s size, and current organizational circumstances (e.g., reorganization).

### Fidelity trajectories and patients’ outcomes

The results showed no significant differences in changes in patient outcomes between the stable high fidelity and moderate and improving fidelity organizations. This is in contrast with the review of Durlak and DuPre (2008) [18]. A possible explanation is that the fidelity scores in our study reflect the extent to which the RSE program was integrated into the routines of the organization according to its predefined program components rather than the extent to which the program components were actually received by individual patients. Interestingly, the data showed that stable high fidelity organizations achieved higher levels of routinization of the program components, which might result in differences between the clusters of organizations in health behavior outcomes of future patients, in favor of the stable high fidelity organizations. Similarly, it is also possible that the lack of a difference between both clusters of organizations can be explained by the fact that we only measured physical activity behavior in a select sample of patients reached by the RSE program. Since the stable high fidelity organizations implemented the program in a more structured way, it is possible that if we had physical activity data from all outpatient rehabilitation patients treated in the organizations, patient-level outcomes might be different between stable high fidelity and moderate and improving fidelity organizations. Our findings clearly illustrate the complexity of conducting multisource research on organizational-level implementation fidelity trajectories and revealing its relationship with patient-level outcomes in a multidisciplinary healthcare.

### Strengths and limitations

The study’s major strength is its longitudinal design including multisource data (organization, professionals and patients) based on mixed methods (quantitative and qualitative). We collected detailed information about the implementation process within each participating organization from different perspectives (managers, physicians, project leaders, counselors) creating a rich and unique dataset. As a result, we were able to apply triangulation techniques making our findings relevant for both implementation research and practice.

The study’s procedure for calculating implementation fidelity has both merits and limitations. The measurement of the total fidelity scores at the organizational level enabled the identification of variety in implementation fidelity trajectories. Clustering of the emerging trajectories enabled the exploration of associations with both the organizational and the aggregated professional- and patient-level factors, following a multiple case study logic [23].

As to the limitations, firstly, the measurement method for these fidelity scores relied on self-constructed items. Moreover, in calculating the total fidelity scores we dichotomized each item and weighed each item equally, but we have no way of knowing whether each item is equally important. Further research is necessary to gain more insight into the reliability and validity of this measurement method.

A related limitation concerns the influence of missing items on the total fidelity scores. Any missing item was conservatively counted as ‘zero,’ which means that the observed fidelity scores may have been somewhat lower than the real scores. This conservative measure seems legitimate: to the extent that an organizations’ professionals do not participate in the program’s evaluation, they can be regarded as less engaged with the program.

A final limitation is that the third cluster’s small sample size (*n* = 2) prohibited an analysis of its characteristics. Nevertheless, the deviating fidelity trajectory in these two organizations reflects real-world phenomena that deserve further study. The steep decrease and increase in fidelity scores over time underscore the dynamic complexity of implementing programs in multidisciplinary settings.

## Conclusions

This study demonstrates a new approach for gaining insight into the heterogeneity of implementation fidelity trajectories of health promotion programs in multidisciplinary settings. The organizational-level implementation fidelity trajectories did not result in outcome differences at patient-level. This may suggest that an effective implementation fidelity trajectory is contingent on the local organization’s conditions. More specifically, achieving stable high implementation fidelity required the management of tensions: realizing a localized change vision, while safeguarding the program’s standardized core components and engaging the scarce physicians throughout the process. When scaling up evidence-informed health promotion programs, we propose to tailor the management of implementation tensions to local organizations’ starting position, size, and circumstances.

### Additional files

Additional file 1: Data fidelity scoresR2. (XLSX 9 kb)

## Appendix 1

**Table 7:** Professionals’ role and the interview design of the conducted interviews (*n* = 23)

Interview	Professionals’ role	Interview design
1	Project leader and manager	Single
2	Counselor	Single
3	Project leader (previous) Project leader (current)	Double
4	Counselor	Single
5	Project leader	Single
6	Project leader and manager Project leader and counselor	Double
7	Project leader Counselor	Double
8	Project leader and manager	Single
9	Project leader and counselor	Single
10	Project leader and manager	Single
11	Project leader and manager	Single
12	Counselors (*n* = 2)	Double
13	Project leader	Single
14	Project leader	Single
15	Project leader and manager	Single
16	Project leader and counselor	Single
17	Project leader and counselor	Single
18	Project leader	Single
19	Counselor	Single
20	Project leader (previous) Project leader (current)	Double
21	Project leader Counselor	Double
22	Program coordinators (*n* = 2)	Double
23	Program coordinators (*n* = 2)	Double

Note. Each line of the table indicates a description of one interview. A total of 23 interviews were conducted, of which 21 interviews were conducted with rehabilitation professionals (*n* = 27) and 2 interviews with program coordinators (*n* = 2). Some rehabilitation professionals fulfilled a combined role, such as project leader and manager or project leader and counselor. The interview design was single (with one professional) or double (with two professionals). The table contains the interviews conducted with professionals working in one of the participating organizations (*n* = 17)

## Appendix 2

**Table 8:** Fidelity components per cluster at the start and at the end of the implementation period

Characteristics	Cluster 1 Stable high fidelity n = 9		Cluster 2 Moderate and improving fidelity n = 6	
Start of implementation period (T0)				
Fidelity components % (n)	Yes	n/a or mv	Yes	n/a or mv
1a. Intake session takes place	89% (8)	–	17% (1)	17% (1)
1b. Intake session standard component	33% (3)	–	17% (1)	17% (1)
2a. Sport and exercise is part of the official policy	44% (4)	–	50% (3)	17% (1)
2b. Discussion during multidisciplinary team	67% (6)	–	33% (2)	50% (3)
3a. Referral to SCC takes place	100% (9)	–	67% (4)	–
3b. Referral to SCC standard component	89% (8)	–	33% (2)	17% (1)
4a. Consultation at SCC takes place	78% (7)	–	83% (5)	–
4b. MI is used by all counselors	44% (4)	–	17% (1)	
5a. Counseling takes place	67% (6)	–	17% (1)	–
5b. Counseling as standard component	67% (6)	–	0	17% (1)
6a. Collaboration with external sport and exercise providers	78% (7)	22% (2)	17% (1)	33% (2)
6b. Network	56% (5)	33% (3)	50% (3)	50% (3)
End of implementation period (T2)				
Fidelity components % (n)	Yes	n/a or mv	Yes	n/a or mv
1a. Intake session takes place	78% (7)	–	67% (4)	–
1b. Intake session standard component	22% (2)	–	67% (4)	–
2a. Sport and exercise is part of the official policy	67% (6)	11% (1)	17% (1)	50% (3)
2b. Discussion during multidisciplinary team	44% (4)	22% (2)	17% (1)	33% (2)
3a. Referral to SCC takes place	100% (9)	–	67% (4)	–
3b. Referral to SCC standard component	78% (7)	–	33% (2)	17% (1)
4a. Consultation at SCC standard component	78% (7)	–	17% (1)	17% (1)
4b. MI is used by all counselors	67% (6)	–	83% (5)	–
5a. Counseling according to the guidelines	56% (5)	22% (2)	17% (1)	33% (2)
5b. Counseling as standard component	100% (9)	–	33% (2)	–
6a. Collaboration with external sport and exercise providers	78% (7)	–	83% (5)	–
6b. Network	78% (7)	11% (1)	83% (5)	

Note. *SCC* Sports Counseling Center, *MI* motivational interviewing, *n/a* not applicable, *mv* missing value

## Appendix 3

**Table 9:** Summary of identified tensions and implementation fidelity trajectories

Tensions	Cluster 1 Stable high fidelity	Cluster 2 Moderate and improving fidelity
1. Organization’s vision and program’s fidelity	Organization’s vision focused - Mainly high or moderate starting positions - Generally more experienced at start	Program’s fidelity focused - Mainly low starting positions
2. Active engagement of physicians and buffering physicians’ engagement	Active engagement of physicians - Mainly small organizations - Mainly stable organizational circumstances	Buffering physicians’ engagement - Mainly large organizations - Relatively more reorganizations
3. High fidelity and cost-efficient implementation	High fidelity implementation - Stable/linear trajectory - Mainly high or moderate starting position - Mainly small organizations	Cost-efficient implementation - Developmental/incremental trajectory - Mainly low starting positions - Mainly large organizations

## Abbreviations

MI: Motivational interviewing
ReSpAct: Rehabilitation, Sports and Active Lifestyle
RSE: Rehabilitation, Sports and Exercise
SCC: Sports Counseling Center
SQUASH: Short QUestionnaire to ASses Health enhancing physical activity

## Footnotes

1 This study takes an organizational-level perspective on implementation fidelity of a health promotion program. In our study, higher implementation fidelity scores represent a more complete implementation of the program’s core components. We label this ‘organizational-level implementation fidelity’. An implementation fidelity trajectory refers to a sequence of implementation fidelity scores measured at subsequent moments in time.
